# The effect of secure attachment on family relationships and peer bullying in adolescents: the mediating role of positive childhood experiences

**DOI:** 10.3389/fpsyg.2025.1700648

**Published:** 2025-11-12

**Authors:** İlhan Çiçek, Zafer Korkmaz, Fırat Ünsal, Zainab Shalal Alanazi, Juan Gómez-Salgado, Murat Yıldırım

**Affiliations:** 1 Child Development Department, Health College, Batman University, Batman, Türkiye; 2 Faculty of Education, Van Yüzüncü Yıl University, Van, Türkiye; 3 Department of Psychology, Faculty of Science and Arts, Bitlis Eren University, Bitlis, Türkiye; 4 Department of Psychology, Shaqra University, Riyadh, Saudi Arabia; 5 Department of Sociology, Social Work and Public Health, Faculty of Labour Sciences, University of Huelva, Huelva, Spain; 6 Safety and Health Postgraduate Program, Universidad Espíritu Santo, Guayaquil, Ecuador; 7 Department of Psychology, Faculty of Science and Letters, Agri Ibrahim Cecen University, Ağrı, Türkiye; 8 Psychology Research Center, Khazar University, Baku, Azerbaijan

**Keywords:** secure attachment, positive childhood experiences, family cohesion, family conflict, peer bullying, adolescent adjustment

## Abstract

**Aims:**

This study examines whether positive childhood experiences (PCEs) mediate the relationship between secure attachment and family conflict, peer bullying, and family cohesion in adolescents.

**Method:**

The sample includes 574 high school students [301 female (52.4%), 273 male], aged 14 to 18 years (*M* = 16.28, *SD* = 1.45). Participants completed the Brief Family Relationships Scale, the Attachment Styles Scale, the Peer Bullying Scale, and the Positive Childhood Experiences Scale.

**Results:**

Findings revealed that secure attachment was positively associated with PCEs, which in turn were linked to greater family cohesion and lower levels of family conflict and peer bullying. Mediation analyses confirmed that PCEs significantly mediated the relationship between secure attachment and family conflict, peer bullying, and family cohesion.

**Conclusion:**

These findings suggest PCEs as a key variable linking secure attachment to adolescents’ social and family adjustment. They emphasize the critical role of nurturing supportive developmental environments across diverse contexts.

## Introduction

Attachment theory constitutes one of the most seminal frameworks in human developmental science. It posits that the earliest relationships established between infants and their primary caregivers—those responsible for meeting fundamental survival and emotional needs—exert profound and enduring influences across the life course. Conceptualized by John Bowlby in the 1960s (Bretherton, 1992), the theory advances the premise that children are endowed with an innate attachment drive (Bowlby, 2013), a biologically adaptive system that functions to secure proximity to caregivers to ensure survival, protection, and optimal socio-emotional growth. The empirical elaborations of Mary Ainsworth (Bretherton, 1992) refined this model through systematic observation, delineating four prototypical attachment styles—secure, anxious-ambivalent, avoidant, and disorganized (Delgado et al., 2022). Among these, secure attachment emerges when caregivers are consistently responsive and accessible, enabling children to internalize expectations of safety, reliability, and worthiness of care (Güvendeğer Doksat and Demirci Ciftci, 2016). These early experiences become inscribed within internal working models that serve as cognitive-affective templates for future relational functioning across the lifespan. Importantly, secure attachment also lays the groundwork for positive childhood experiences (PCEs), defined as supportive, safe, and nurturing interactions that promote wellbeing and adaptive relational development (Chris Fraley, 2002).

Adolescence represents a pivotal developmental juncture wherein attachment relationships undergo substantial reconfiguration. This period is marked by a transition from childhood dependence toward adult autonomy, characterized by reductions in physical proximity and overt parental reliance (Delgado et al., 2022). Importantly, however, this process does not dissolve attachment bonds; rather, securely attached adolescents continue to derive a sense of assurance from the perceived availability and emotional accessibility of their parents (Güvendeğer Doksat and Demirci Ciftci, 2016). The restructuring of attachment in adolescence is guided by the enduring internal working models formed in early childhood, which in turn foster PCEs that are linked with social functioning and peer relational patterns (Delgado et al., 2022). Empirical evidence indicates that adolescents with secure attachment tend to report higher levels of PCEs, which are associated with more adaptive interactions within family and peer contexts (Cassidy and Shaver, 2018; Keizer et al., 2019). Moreover, secure attachment functions as a protective factor, attenuating risk for depression (Qu et al., 2025; Spruit et al., 2020), enhancing self-esteem (Keizer et al., 2019), and reducing vulnerability to anxiety, mood disturbances, and behavioral dysregulation through its facilitative role in emotion regulation and stress coping (Auxilia and Mishra, 2024; Kokkinos et al., 2019). Secure attachment also predicts the development of adaptive peer relationships (Delgado et al., 2022), while the continuity of early attachment experiences into adulthood has been robustly demonstrated (Chris Fraley, 2002). Emerging findings further highlight that family communication patterns during adolescence critically mediate these processes (Blake et al., 2024), and that parent-child attachment significantly influences adolescent socio-emotional adjustment through its effects on emotion regulation (Wang et al., 2024). Collectively, these findings demonstrate the urgent need for attachment-based preventive and therapeutic interventions.

Within the familial domain, the constructs of family cohesion and family conflict represent critical dimensions of relational functioning. Family cohesion refers to the strength of emotional connectedness, mutual support, and satisfaction within the family system, whereas family conflict reflects recurrent patterns of discord, tension, and incompatibility (Çiçek and Yıldırım, 2025; Kars and Peker, 2025). Both constructs are deeply implicated in shaping psychosocial development and systemic functioning (Roman et al., 2025). Secure attachment provides the conditions that foster PCEs, such as open communication and emotional reciprocity, which are in turn associated with higher family cohesion and lower family conflict. Multiple studies emphasize that secure attachment is a cornerstone for the cultivation of adaptive family processes and harmonious relational climates (Kars and Peker, 2025; Roman et al., 2025; Vegas, 2025).

Secure attachment, by encouraging a firm belief that one is seen as lovable and valued (Eilert and Buchheim, 2023), facilitates openness, emotional reciprocity, and trust within family systems. In doing so, it enhances family satisfaction, resilience, and functional cohesion (Roman et al., 2025; Vegas, 2025). Multiple studies emphasize that secure attachment is a cornerstone for the cultivation of adaptive family processes and harmonious relational climates (Roman et al., 2025; Vegas, 2025). Importantly, secure attachment also plays a protective role in conflict management: securely attached individuals exhibit reduced emotional reactivity, greater emotional regulation, and employ more constructive conflict resolution strategies (Biglan et al., 2015; Rahal and Fosco, 2025). As Kohlhoff et al. (2022) highlights, secure attachment fosters the establishment of healthy boundaries and transforms conflicts into opportunities for mutual understanding and relational strengthening.

Beyond normative family functioning, secure attachment also confers resilience under adverse external conditions. Research indicates that secure attachment bolsters family cohesion when confronted with external risk factors such as economic stress or cultural pressures (Davila et al., 2025; Galaitsi et al., 2024). Secure attachment enhances psychological resilience, enabling families to sustain adaptive functioning even under duress. The quality of parent-child interactions, particularly in securely attached dyads, serves as a critical determinant of family cohesion (Eilert and Buchheim, 2023). Adolescents embedded in cohesive families demonstrate greater capacity to tolerate depressive symptoms, even in contexts of parental insensitivity (Yu et al., 2025).

By contrast, insecure attachment styles—whether anxious, avoidant, or disorganised—amplify familial discord, particularly during adolescence, and are associated with maladaptive outcomes including experiential avoidance, emotional dysregulation, and self-harming behaviors (Li, 2025; Pu et al., 2025; Zhang et al., 2023). Evidence suggests a bidirectional, self-reinforcing cycle: family conflict fosters insecure attachment, while insecure attachment intensifies and perpetuates conflict (Joo and Lee, 2020; Neville et al., 2025). Attachment-based interventions offer a potential pathway to disrupt this cycle by cultivating secure relational strategies and constructive conflict management (Kohlhoff et al., 2022).

Adolescence also constitutes a developmental period in which peer bullying emerges as a salient psychosocial threat. Secure attachment appears to function as a protective buffer against both victimization and perpetration. Secure parental relationships promote emotional regulation, reduce aggression, boost self-esteem, and equip adolescents with effective strategies for navigating peer conflict (Charalampous et al., 2018; Lin et al., 2022; Özada and Duyan, 2018). Open communication and parental accessibility facilitate constructive peer conflict resolution, thereby disrupting cycles of bullying (Carter et al., 2023; Yılmaz, 2024). Empirical findings confirm that securely attached adolescents report heightened perceptions of social support and markedly lower involvement in bullying (Coşkun et al., 2024). Such protective effects also extend to buffering depressive and trauma-related sequelae associated with bullying by enhancing resilience (Lin et al., 2022; Mahmood et al., 2025).

Conversely, insecure attachment styles increase susceptibility to both bullying victimization and perpetration (Mahmood et al., 2025; Williams, 2011). Adolescents with weak parental bonds frequently exhibit either heightened aggression or passive acquiescence in peer contexts (Özen and Aktan, 2010; Yöndem and Totan, 2007). These patterns, often mediated by low self-esteem and deficient social skills (Rokach and Clayton, 2023), intensify depressive, anxious, and traumatic outcomes (Yavrutürk, 2025). Moreover, inadequate parental support and fragile peer bonds exacerbate these effects, leading to chronic psychopathology and maladaptive behaviors such as self-harm or school refusal (Charalampous et al., 2018; Coşkun et al., 2024; Yavrutürk, 2025). Consequently, interventions targeting parent-adolescent attachment should be considered a critical axis of bullying prevention and mental health promotion (Carter et al., 2023; Özada and Duyan, 2018).

Taken together, these findings suggest that secure attachment is indirectly associated with adolescents’ peer and family outcomes through its influence on PCEs. Accordingly, the present study examines whether PCEs mediate the relationship between secure attachment and adolescent adjustment.

### Mediation role of PCEs

Secure attachment is a fundamental psychological mechanism that arises when a child forms a consistent, responsive, and accessible bond with the carer, and it is considered essential for fostering PCEs (Bowlby, 2013; Cassidy and Shaver, 2018). Within this developmental context, secure attachment fostered through PCEs functions as a protective buffer against a wide spectrum of maladaptive outcomes, including depression, anxiety, and behavioral dysregulation across adolescence and adulthood (Lewis and McKelvey, 2025; Şanli et al., 2024; Scholtes and Cederbaum, 2024). Empirical evidence underscores that such experiences extend beyond immediate developmental benefits; they enhance psychological resilience even into later stages of life (Qu et al., 2025), attenuate internalizing and externalizing symptomatology (Choi et al., 2024), and substantially improve perceived quality of life (Luo et al., 2022).

A growing body of literature highlights that PCEs exert profound salutogenic effects on adolescent mental health trajectories, fostering not only resilience but also adaptive emotional regulation capacities (Choi et al., 2024; Lewis and McKelvey, 2025). Importantly, these experiences demonstrate a compensatory capacity, mitigating the deleterious sequelae of adverse childhood experiences (ACEs) (Qu et al., 2025; Scholtes and Cederbaum, 2024). Protective factors such as a cohesive and supportive family climate, the cultivation of positive peer relations, and the acquisition of robust social competencies serve as mediating mechanisms that enhance adolescents’ wellbeing, reduce vulnerability to psychopathology, and reinforce trajectories of healthy psychosocial adjustment (Auxilia and Mishra, 2024; Luo et al., 2022).

Through these pathways, PCEs enable adolescents to navigate developmental transitions with greater efficacy by strengthening their capacity for social adjustment, adaptive coping, and complex problem-solving (Crandall et al., 2020). Accordingly, PCEs can be conceptualized as a mediating nexus that not only facilitates resilience in the face of normative developmental stressors but also stabilizes family relational dynamics. Collectively, the extant evidence suggests that secure attachment—when scaffolded by PCEs—exerts a dual influence: promoting balance within family relationships during adolescence and serving as a protective factor against vulnerability to peer bullying victimization. Moreover, PCEs appear to play a mediational role in these processes, bridging early attachment experiences with later adolescent psychosocial outcomes.

### Current study

This study examines the effects of secure attachment on adolescents’ family relational dynamics, focusing on conflict, cohesion, and experiences of peer bullying. This study also seeks to clarify the protective role of positive childhood experiences (PCEs) in influencing psychological and social outcomes. This research posits that PCEs have a positive impact on adolescent development by promoting secure attachment patterns, which subsequently improve family cohesion, decrease familial conflict, and lower susceptibility to peer bullying.

The originality of this study lies in its integrative examination of these interrelated domains. While the extant literature has largely addressed attachment, family functioning, and peer bullying as distinct phenomena (Charalampous et al., 2018; Delgado et al., 2022; Mahmood et al., 2025; Özen and Aktan, 2010), the present research introduces a holistic framework that considers their dynamic interplay. More specifically, it advances the novel proposition that secure attachment may serve as a mechanism linking PCEs to enhanced family cohesion and adaptive coping in the context of peer bullying. By articulating this integrative model, the study extends the empirical base of attachment theory and offers new insights for preventive and intervention-oriented frameworks.

This contribution is of particular value in the Turkish setting, where empirical research exploring the concurrent influences of secure attachment and PCEs on family cohesion and bullying-related coping remains scarce. In addressing this lacuna, the present study not only provides a culturally grounded exploration of these characteristics but also proposes a unique evidence base for building attachment-based and resilience-promoting treatments for adolescents.

In accordance with this theoretical framework, three assumptions are advanced:

*H1*. Secure attachment will significantly and negatively predict family conflict and peer bullying, while positively predicting family cohesion.

*H2*. PCEs will significantly and negatively predict family conflict and peer bullying, while positively predicting family cohesion.

*H3*. PCEs will mediate the relationship between secure attachment and family conflict, peer bullying, and family cohesion.

**Figure 1:**
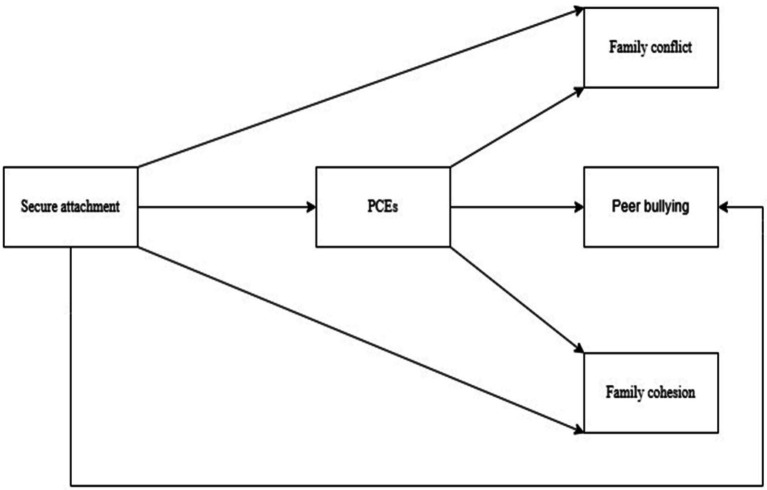
The hypothesized research model.

To empirically test these hypotheses, a mediation model was specified, enabling the examination of both direct and indirect associations among the focal variables. The hypothesized mediation model is presented in Figure 1.

## Method

### Participants and procedures

The data for this study, which employed a convenience sampling method, were collected from adolescents enrolled in secondary schools in Turkey between April 10 and May 5, 2025. The sample comprised 574 students in total, including 301 girls (52.4%) and 273 boys, aged between 14 and 18 years (*M* = 16.28, *SD* = 1.45). Inclusion criteria required participants to be enrolled in secondary school, aged 14–18, and able to provide informed consent. Exclusion criteria included students with diagnosed severe cognitive or developmental disorders that could impair their ability to complete the questionnaires, as well as those who did not provide consent. Ethical approval for the study was obtained from the Ethics Committee of *[Blinded for review]* University (Ethics Code: 2025/04–11, E.7252), and all procedures were carried out in accordance with the principles of the Helsinki Declaration. Before administering the questionnaires, participants were provided with comprehensive information regarding the study’s purpose, the confidentiality of their responses, and the specific use of the collected data. Informed consent was obtained from all participants, and no financial or material compensation was offered for participation.

### Measures

#### The Brief Family Relationship Scale (BFRS)

The scale was developed by Fok et al. (2014), was designed to assess adolescents’ perceptions of family functioning. The BFRS is a 16-item self-report measure that evaluates three dimensions of family relationships: family cohesion (seven items), family expressiveness (three items), and family conflict (six items). Responses are given on a 5-point Likert-type scale ranging from 1 (very rarely) to 5 (very often). In the present study, only the family conflict and family cohesion subscales were utilized. Higher scores on the family conflict subscale indicate more frequent conflicts, whereas higher scores on the family cohesion subscale reflect more balanced and harmonious family relationships. The Turkish adaptation of the scale was conducted by Yıldırım et al. (2023). In the current study, Cronbach’s alpha coefficients were *α* = 0.90 for family conflict and *α* = 0.77 for family cohesion.

#### Peer Bullying Scale (PBS)

In the present study, we employed the Peer Bullying Scale (PBS), developed by Shaw et al. (2013), to assess adolescents’ involvement in bullying behaviors. The scale comprises 10 items rated on a 5-point Likert-type scale (1 = never done it, 2 = done it once or twice, 3 = done it once a month, 4 = done it once a week, 5 = done it several times a week). The scale does not include any reverse-coded items. Total scores range from 10 to 50, with higher scores indicating greater engagement in peer bullying behaviors. The Turkish adaptation of the scale was conducted by Arslan (2017). In the current study, the scale demonstrated high internal consistency, with a Cronbach’s alpha coefficient of α = 0.90.

#### Positive childhood experiences scores (PCEs)

The scale, developed by Bethell et al. (2019), comprises a single dimension with seven items. Items are rated on a 5-point Likert-type scale (1 = Never, 5 = Always), and the scale contains no reverse-scored items. Total scores range from 7 to 35, with higher scores reflecting a higher number of positive experiences during childhood, prior to the age of 18. The Turkish adaptation of the scale was conducted by Çi̇çek and Çeri̇ (2021). In the present study, the scale demonstrated satisfactory internal consistency, with a Cronbach’s alpha coefficient of *α* = 0.84.

#### Attachment Styles Scale Short Form (ASS-SF)

The scale, originally developed by Feeney et al. (1994) and shortened by Iwanaga et al. (2018), comprises three subscales: secure, anxious, and avoidant attachment, with each subscale containing four items. The scale assesses individuals’ attachment styles with respect to their parents using a 6-point Likert-type format. The Turkish adaptation of the ASS-SF was conducted by Çelik (2024). In the present study, only the secure attachmen*t* subscale was utilized. Internal consistency analysis revealed a Cronbach’s alpha coefficient of *α* = 0.76 for this subscale.

## Results

### Data analysis

Data analysis for the present study was conducted using IBM SPSS Statistics (version 25), with mediation analyses performed via the PROCESS macro (Model 4) (Hayes, 2022). The analytical procedure commenced with descriptive statistics, including means, standard deviations, skewness, and kurtosis, to evaluate the normality of the variable distributions. Pearson correlation coefficients were then calculated to examine the bivariate relationships among the study variables. The internal consistency of each scale was assessed using Cronbach’s alpha coefficients, confirming the reliability of the measures within the current sample. Simple mediation models were tested using PROCESS Model 4 (Hayes, 2022). PCEs served as the single mediator in each model, with family cohesion, family conflict, and peer bullying analyzed as separate outcomes. Bootstrapping with 5,000 resamples was applied to estimate indirect effects and 95% confidence interval (Hayes, 2018).

### Preliminary analyses

Preliminary analyses indicated that all study variables demonstrated normal distribution, with skewness values ranging from −0.61 to 1.08 and kurtosis values ranging from −0.096 to 1.59, falling within acceptable limits (≤ |2|) (Kline, 2023). Pearson correlation analyses revealed a significant positive association between secure attachment and positive childhood experiences (PCEs) (*r* = 0.44, *p* < 0.01) as well as family cohesion (*r* = 0.41, *p* < 0.01). Conversely, secure attachment was significantly and negatively correlated with family conflict (*r* = −0.26, *p* < 0.01) and peer bullying (*r* = −0.22, *p* < 0.01). PCEs were also significantly negatively associated with family conflict (*r* = −0.40, *p* < 0.01) and peer bullying (*r* = −0.19, *p* < 0.01), while showing a strong positive relationship with family cohesion (*r* = 0.81, *p* < 0.01). Internal consistency reliability coefficients for the instruments ranged from *α* = 0.76 to *α* = 0.90 (see Table 1), indicating satisfactory reliability for the scales employed in the present study.

**Table 1:** Descriptive statistics and correlation matrix.

Variables	1.	2.	3.	4.	5.
1. Secure attachment	1	0.44^**^	−0.26^**^	−0.22^**^	0.41^**^
2. PCEs		1	−0.40^**^	−0.19^**^	0.81^**^
3. Family conflict			1	0.25^**^	−0.53^**^
4. Peer bullying				1	−0.27^**^
5. Family cohesion					1
Mean	15.11	22.15	13.03	16.96	20.54
SD	5.54	6.96	5.44	8.23	6.47
Skewness	−0.230	−0.186	0.464	1.08	−0.615
Kurtosis	−0.587	−0.701	−0.962	1.59	−0.873
Internal reliability (α)	0.76	0.84	0.90	0.90	0.77

**All correlation coefficients are significant at the level *p* < 0.001.

### Mediation analysis

**Figure 2:**
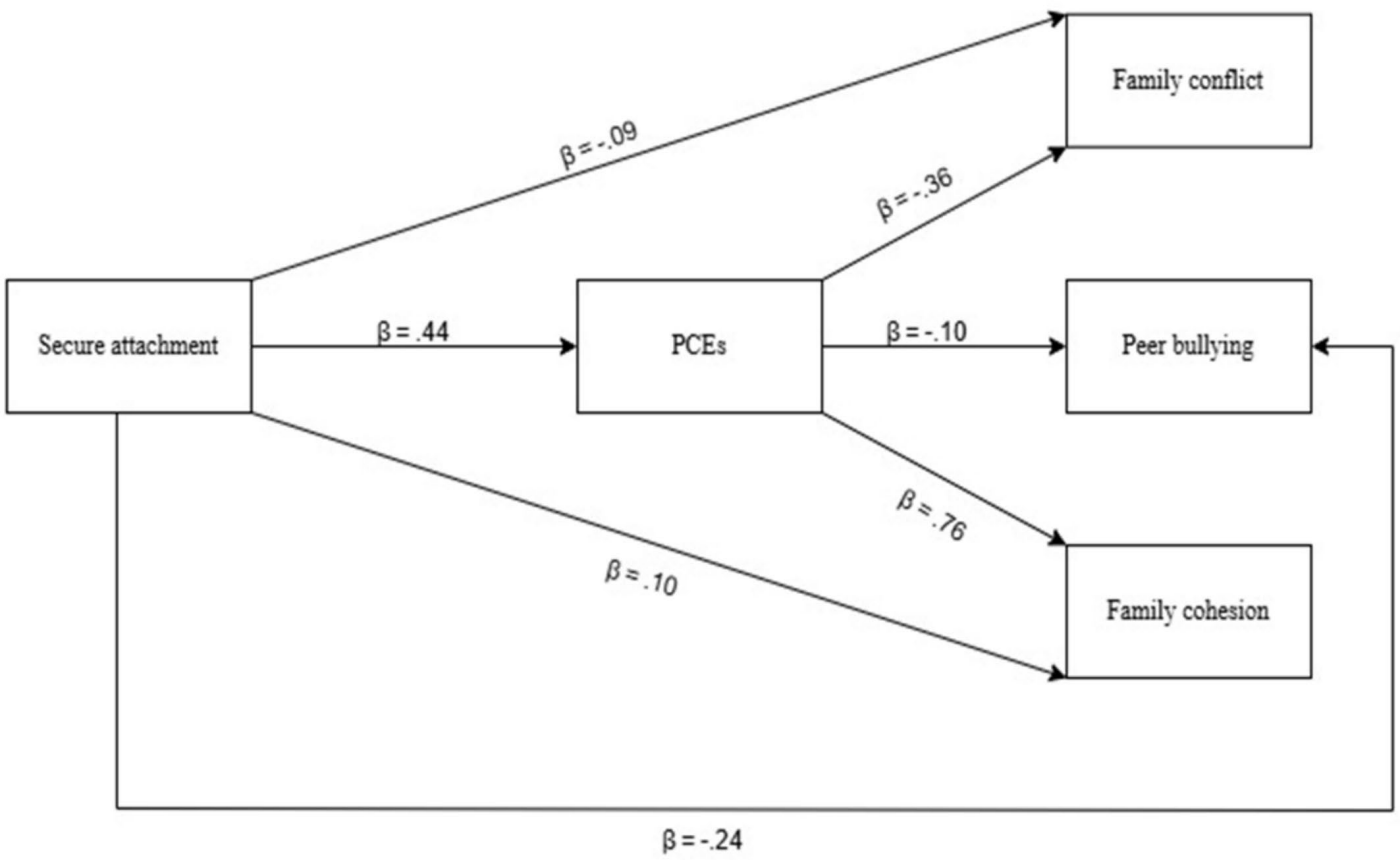
The tested research model.

Following preliminary analyses, simple mediation analyses were conducted to examine the mediating role of positive childhood experiences (PCEs) in the relationships between secure attachment and family conflict, peer bullying, and family cohesion (Figure 2; Tables 2, 3). Results indicated that secure attachment positively and significantly predicted PCEs (β = 0.44, *p* < 0.001), accounting for 19% of the variance in PCEs. Secure attachment also demonstrated a significant negative association with family conflict (β = −0.09, *p* < 0.001) and peer bullying (β = −0.24, *p* < 0.001), while positively predicting family cohesion (β = 0.10, *p* < 0.001). When considered alongside PCEs, secure attachment explained 17% of the variance in family conflict, 5% in peer bullying, and 65% in family cohesion.

**Table 2:** Unstandardized coefficients for the proposed mediation model.

Antecedent	Consequent			
	*M* (PCEs)			
	Coeff.	*SE*	*t*	*p*
*X* (Secure attachment)	0.670	0.058	11.55	<0.001
Constant	12.033	0.922	13.05	<0.001
	*R*^2^ = 0.19			
	*F* = 135.08; *p* < 0.001			
	*Y_1_* (Family conflict)			
*X* (Secure attachment)	−0.119	0.050	−2.34	<0.001
*M* (PCEs)	−0.281	0.033	−8.48	<0.001
Constant	21.111	0.823	25.62	<0.001
	*R*^2^ = 0.17			
	*F* = 58.80; *p* < 0.001			
	*Y_2_* (Peer bullying)			
*X* (Secure attachment)	−0.304	0.082	−3.67	<0.001
*M* (PCEs)	−0.137	0.053	−2.55	<0.01
Constant	24.65	1.34	18.35	<0.001
	*R*^2^ = 0.05			
	*F* = 17.42; *p* < 0.001			
	*Y_3_* (Family cohesion)			
*X* (Secure attachment)	0.102	0.039	2.60	<0.01
*M* (PCEs)	0.717	0.025	27.90	<0.001
Constant	3.064	0.638	4.80	<0.001
	*R*^2^ = 0.65			
	*F* = 525.80; *p* < 0.001			

Number of bootstrap samples for percentile bootstrap confidence intervals: 5,000. SE, standard error; Coeff, unstandardized coefficient; X, independent variable; M, mediator variable; Y, outcome variable.

**Table 3:** Mediation model path analysis.

Path	Effect size	SE	95% confidence interval	
			Lower limit	Upper limit
Direct effect				
Secure attachment → family conflict	−0.119	0.050	−0.219	−0.019
Secure attachment → peer bullying	−0.304	0.082	−0.467	−0.141
Secure attachment → family cohesion	0.102	0.039	0.025	0.180
Indirect effect				
Secure attachment → PCEs → family conflict	−0.158	0.026	−0.213	−0.108
Secure attachment → PCEs → peer bullying	−0.050	0.021	−0.096	−0.012
Secure attachment → PCEs → family cohesion	0.338	0.030	0.278	0.398
Total effect				
Secure attachment → family conflict	−0.308	0.048	−0.404	−0.213
Secure attachment → peer bullying	−0.396	0.074	−0.544	−0.249
Secure attachment → family cohesion	0.588	0.054	0.478	0.692

Importantly, PCEs were found to mediate the associations between secure attachment and all three outcomes. The indirect effects were significant for family conflict [effect = −0.15, 95% CI (−0.21, −0.10)], peer bullying [effect = −0.050, 95% CI (−0.09, −0.01)], and family cohesion [effect = 0.33, 95% CI (0.27, 0.39)]. Moreover, both the direct and total effects of secure attachment on these outcomes remained significant, indicating partial mediation (see Table 3). These findings underscore the pivotal role of PCEs as a protective mechanism through which secure attachment contributes to more harmonious family relationships and reduced engagement in peer bullying.

## Discussion

The present study aimed to investigate the extent to which secure attachment was associated with lower levels of family conflict and peer bullying during adolescence while being positively associated with family cohesion. Grounded in attachment theory, secure relationships established with parents during childhood are posited to foster positive internal working models of the self and others (Keizer et al., 2019; Sevi̇nç and Şener Kilinç, 2018), which in turn may be linked social interactions and relational outcomes during adolescence (Delgado et al., 2022).

The study’s first hypothesis (H1)—that secure attachment would show negative associations with family conflict and peer bullying while being positively associated with family cohesion—was strongly supported. This finding underscores the relevance of secure attachment in relation to both individual well-being and family functioning. Specifically, the negative association between secure attachment and family conflict aligns with prior literature emphasizing that securely attached individuals tend to demonstrate enhanced emotion regulation and conflict management capacities, which are associated with reduced emotional reactivity during familial disagreements (Biglan et al., 2015; Keizer et al., 2019; Keskin and Çam, 2008; Perron, 2013; Rahal and Fosco, 2025; Roman et al., 2025).

Similarly, secure attachment was found to be significantly and negatively associated with peer bullying, corroborating evidence that adolescents with secure parent-child relationships are less likely to be involved in bullying (Charalampous et al., 2018; Lin et al., 2022; Özada and Duyan, 2018). Securely attached adolescents typically report stronger social skills and broader social support networks, enabling them to navigate peer interactions more effectively and maintain higher self-esteem, thereby being less involved in reducing the risk of involvement in bullying dynamics (Carter et al., 2023; Coşkun et al., 2024; Özen and Aktan, 2010; Williams, 2011).

The positive association of secure attachment on family cohesion further confirmed H1. Secure attachment appears to be related to emotional closeness, mutual support, and satisfaction within family systems (Kars and Peker, 2025; Roman et al., 2025; Vegas, 2025). This finding resonates with existing research indicating that secure attachment is linked to familial warmth, trust, and collective resilience in the face of external stressors (Cassidy and Shaver, 2018; Davila et al., 2025; Galaitsi et al., 2024; Sevi̇nç and Şener Kilinç, 2018; Spruit et al., 2020). It is, however, critical to acknowledge that family cohesion is also shaped by environmental and experiential factors, including PCEs (Blake et al., 2024; Wang et al., 2024).

We also strongly supported the study’s second hypothesis (H2), which states that PCEs would be negatively associated with family conflict and peer bullying while being positively associated with family cohesion. These findings align with research emphasizing the importance of protective and promotive factors as alternatives to trauma-focused models (Bethell et al., 2019; Crandall et al., 2020; Scholtes and Cederbaum, 2024). Specifically, adolescents reporting higher PCEs also reported lower family conflict (Li, 2025; Neville et al., 2025; Pu et al., 2025), lower peer bullying (Mahmood et al., 2025; Yavrutürk, 2025; Zhang et al., 2023), and higher family cohesion (Davila et al., 2025; Roman et al., 2025; Vegas, 2025).

The negative association between PCEs and family conflict indicates that experiences such as supportive caregiving, a secure family environment, and a sense of belonging may be linked to enhanced adolescents’ coping and interpersonal problem-solving skills. These capacities are related to lower escalation of typical adolescent disagreements into destructive conflicts (Pu et al., 2025). Correspondingly, the strong positive association between PCEs and family cohesion underscores that family cohesion may be linked not merely to conflict avoidance but through actively cultivated positive experiences characterized by warmth, trust, and emotional support (Davila et al., 2025; Galaitsi et al., 2024; Roman et al., 2025).

With respect to peer bullying, PCEs showed a significant negative association, suggesting their relevance extends beyond familial contexts. Mechanistically, PCEs promote psychological resilience, social and emotional skill development, and mental wellbeing, thereby being linked to lower involvement in the adverse impacts of peer victimization (Elmore and Crouch, 2020; Lin et al., 2022; Luo et al., 2022; Qu et al., 2022). These findings reinforce the notion that psychosocial interventions should focus not only on reducing risk factors but also on enhancing protective experiences, particularly in adolescence.

The study’s third hypothesis (H3)—clarified that secure attachment functions as a key mediator linking PCEs to adolescent outcomes. Positive childhood experiences were significantly associated with higher secure attachment, which in turn predicted lower family conflict and peer bullying and greater family cohesion. These findings highlight secure attachment as a mechanism through which early positive experiences exert protective effects on adolescent social and familial adjustment. This aligns with longitudinal and cross-cultural evidence indicating that supportive early experiences strengthen attachment security, which fosters adaptive coping, effective communication, and conflict resolution skills in adolescence (Akbulut, 2025; Biglan et al., 2015; Davila et al., 2025; Lewis and McKelvey, 2025; Roman et al., 2025).

Although both secure attachment and PCEs were negatively associated with peer bullying, the association of PCEs was weaker than that of secure attachment. The difference may reflect the limited link of PCEs on the perpetrator dimension of bullying (Scholtes and Cederbaum, 2024), the increasing salience of peer norms during adolescence (Carter et al., 2023), or measurement limitations in differentiating bully, victim, and bully-victim roles (Coyle et al., 2021). Nonetheless, PCEs remain a critical mediating factor, indirectly associated with adolescent psychosocial outcomes by reinforcing secure attachment and supporting adaptive coping in family and peer contexts.

Collectively, the findings of this study indicate that secure attachment and PCEs are interrelated factors associated with adolescent well-being, family cohesion, and adaptive peer relationships. In collectivist cultures such as Turkey, the association of PCEs on family cohesion may be particularly pronounced, reflecting the importance of relational attachment and social support (Kars and Peker, 2025). From a practical perspective, these findings suggest that attachment-based interventions could benefit from integrating strategies that enhance PCEs, and anti-bullying programs may be strengthened by incorporating attachment- and PCE-focused modules targeting both victims and perpetrators to maximize protective effects.

### Implications

The findings of this study make substantial contributions to both theoretical and practical domains by facilitating a nuanced re-examination of attachment theory within the Turkish cultural context. The observed effects of secure attachment on psychosocial outcomes through PCEs provide empirical support for Bowlby’s concept of internal working models, demonstrating that these cognitive-affective schemas are not fixed but can be shaped and reinforced across the lifespan by positive experiences (Cassidy and Shaver, 2018; Lewis and McKelvey, 2025). This reconceptualization positions attachment as a dynamic, malleable process rather than a static trait.

From a practical perspective, the results underscore the value of implementing parent education programs aimed at fostering PCEs within family-based interventions. In school settings, these findings highlight the importance of structured emotional support and empathy training, targeting both victims and perpetrators of bullying to promote resilience and adaptive peer relationships. Clinically, it is recommended that therapeutic interventions consider both individuals’ attachment histories and levels of PCEs, integrating culturally sensitive approaches that account for extended family involvement, particularly in collectivist contexts such as Turkey.

In sum, this study emphasizes that enhancing adolescent mental health requires a dual approach: mitigating risk factors while actively promoting protective factors, with a particular focus on cultivating PCEs. The mediating role of PCEs in shaping family cohesion and conflict offers a comprehensive lens through which to understand the real-life operation of attachment-based internal working models. These findings strongly advocate for integrating attachment-focused and PCE-oriented strategies in adolescent mental health interventions. Beyond its theoretical contribution, the study fills a critical gap in the literature, providing empirical evidence that attachment and PCEs should be addressed together to optimize psychosocial outcomes among adolescents in Turkey and similar cultural settings.

### Limitations and future research directions

Despite the valuable insights yielded by this study, several methodological and conceptual limitations warrant careful consideration when interpreting the findings. First, the cross-sectional design precludes the establishment of causal relationships among variables. Specifically, the influence of secure attachment on family cohesion via PCEs can only be examined more rigorously through longitudinal or experimental research designs. Second, the study sample, comprising urban high school students aged 14–18, limits the generalizability of the findings to younger children, adults, or adolescents residing in rural areas. Measurement limitations are also noteworthy. For instance, while the Peer Bullying Scale captures the perpetrator role, it does not differentiate between perpetrators and victims, thereby restricting insights into victimization experiences. Additionally, the exclusive reliance on self-report measures increases susceptibility to social desirability bias.

Conceptually, the operationalization of PCEs in this study does not fully capture social resources salient in Turkey’s collectivist culture, such as extended family members and broader community networks (Kars and Peker, 2025). Moreover, neurobiological mechanisms potentially underlying the effects of attachment and PCEs were not examined, and other contextual factors that may influence family dynamics, such as economic stress or parental mental health, were not incorporated into the model.

Future research should address these limitations through more comprehensive and methodologically rigorous designs. Comparative studies examining the mediating role of PCEs in attachment–family cohesion relationships across collectivist and individualistic cultures could illuminate culturally specific dynamics—for example, exploring whether PCEs exert a stronger influence on family cohesion in Asian contexts relative to Western societies. Qualitative research capturing detailed adolescent narratives on positive childhood experiences, such as family rituals or safe exploration spaces, would enrich the understanding of PCEs’ mechanisms. Integrating biological markers, such as cortisol or oxytocin levels, could further elucidate the interplay between neurobiological processes, attachment, and PCEs.

Application-focused research is also recommended. The efficacy of school-based interventions designed to enhance PCEs, such as the Family Stories Workshop, could be evaluated using randomized controlled trials. Furthermore, the development of culturally sensitive PCE measurement tools that incorporate extended family and community relationships in Turkey is necessary. Observational assessments of bullying behaviors and teacher reports should complement self-reported data to improve measurement accuracy. Investigating the protective role of PCEs among at-risk populations, including socioeconomically disadvantaged youth or adolescents with migrant backgrounds, would facilitate tailored intervention programs.

Finally, identifying priority research areas within the Turkish context is essential for culturally informed methodological approaches. For example, survey studies examining rural–urban differences could guide locally relevant programs implemented by civil society organizations. Collectively, these directions will contribute to the theoretical refinement of attachment models and the development of evidence-based, PCE-cantered interventions adapted to cultural and contextual specificities.

## Conclusion

This study investigated the influence of secure attachment on family relationships and peer bullying in adolescents, highlighting the critical mediating role of PCEs in these dynamics. While secure attachment independently contributes to psychosocial well-being, combining PCEs significantly amplifies its protective and promotive effects. Specifically, PCEs mitigate family conflict and peer bullying and enhance the capacity of secure attachment to foster family cohesion, effective conflict resolution, and adaptive coping strategies in social interactions.

A notable contribution of this study is its culturally contextualized perspective. In collectivist societies such as Turkey, where extended family networks play a salient role, PCEs appear to reinforce the impact of secure attachment, demonstrating that the internal working models proposed by attachment theory manifest in observable social and relational outcomes through positive childhood experiences. However, given the cross-sectional design, these associations should not be interpreted as causal, and the directionality of effects cannot be firmly established. Practically, these findings underscore that interventions aimed at promoting adolescent mental health may benefit from considering PCEs and attachment together, though empirical validation through longitudinal or experimental studies is needed before clinical or educational application.

In conclusion, this study provides preliminary empirical evidence supporting the integration of attachment-focused and strengths-based approaches in adolescent development. The study’s demonstration of the synergistic effects of secure attachment and PCEs advocates for a paradigm shift from risk-oriented models to interventions that emphasize protective and promotive factors. Future research is encouraged to explore cross-cultural variations, neurobiological mechanisms underpinning the attachment-PCE–PCE relationship, and the emerging interface between digital attachment experiences and positive childhood experiences. These investigations will clarify how early relational experiences influence psychosocial development in various contexts.

## Data Availability

The raw data supporting the conclusions of this article will be made available by the authors, without undue reservation.

## References

[ref1] Akbulut Ö. F. (2025). Positive childhood experiences, suicide cognitions, subjective happiness, and mental well-being in young adults: a half-longitudinal serial mediation study. Psychiatry Q. doi: 10.1007/s11126-025-10162-6, PMID: 40434585 PMC13518451

[ref2] Arslan N. (2017). Peer bullying among high school students: Turkish version of bullying scale. Turk. Online J. Educ. Technol. 5, 853–857.

[ref3] Auxilia A. A. Mishra H. (2024). Examining the connection between attachment and risk for psychopathology in adolescents: a comprehensive review. Int. J. Multidiscip. Res. 6:24510. doi: 10.36948/ijfmr.2024.v06i04.24510

[ref4] Bethell C. Jones J. Gombojav N. Linkenbach J. Sege R. (2019). Positive childhood experiences and adult mental and relational health in a statewide sample: associations across adverse childhood experiences levels. JAMA Pediatr. 173:e193007. doi: 10.1001/jamapediatrics.2019.3007, PMID: 31498386 PMC6735495

[ref5] Biglan A. Gau J. M. Jones L. B. Hinds E. Rusby J. C. Cody C. . (2015). The role of experiential avoidance in the relationship between family conflict and depression among early adolescents. J. Context. Behav. Sci. 4, 30–36. doi: 10.1016/j.jcbs.2014.12.001

[ref6] Blake J. A. Thomas H. J. Pelecanos A. M. Najman J. M. Scott J. G. (2024). The unique role of adolescent internalizing and externalizing problems, and maternal-adolescent communication in their association with attachment in early adulthood. Acta Psychol. 246:104273. doi: 10.1016/j.actpsy.2024.104273, PMID: 38636402

[ref7] Bowlby J. (2013). Bağlanma: Bağlan ve kaybetme 1: Pinhan Yayıncılık.

[ref8] Bretherton I. (1992). The origins of attachment theory: John Bowlby and Mary Ainsworth. Dev. Psychol. 28, 759–775. doi: 10.1037/0012-1649.28.5.759

[ref9] Carter M. Van Der Watt R. Esterhuyse K. (2023). Parent and peer attachment in bullying experiences among pre-adolescents. J. Psychol. Afr. 33, 26–34. doi: 10.1080/14330237.2023.2182948

[ref10] Cassidy J. Shaver P. R. (2018). Handbook of attachment: theory, research, and clinical applications. 3rd Edn. New York, NY: Guilford Press.

[ref11] Çelik M. (2024). Bağlanma Stilleri Ölçeği Kısa Formunun Psikometrik Özelliklerinin İncelenmesi. J. Hist. School 17, 3121–3141. doi: 10.29228/joh.78675

[ref12] Charalampous K. Demetriou C. Tricha L. Ioannou M. Georgiou S. Nikiforou M. . (2018). The effect of parental style on bullying and cyber bullying behaviors and the mediating role of peer attachment relationships: a longitudinal study. J. Adolesc. 64, 109–123. doi: 10.1016/j.adolescence.2018.02.003, PMID: 29448185

[ref13] Choi K. R. Bravo L. La Charite J. Cardona E. Elliott T. James K. F. . (2024). Associations between positive childhood experiences (PCEs), discrimination, and internalizing/externalizing in pre-adolescents. Acad. Pediatr. 24, 1236–1245. doi: 10.1016/j.acap.2024.07.006, PMID: 39004299 PMC11649072

[ref14] Chris Fraley R. (2002). Attachment stability from infancy to adulthood: meta-analysis and dynamic modeling of developmental mechanisms. Personal. Soc. Psychol. Rev. 6, 123–151. doi: 10.1207/S15327957PSPR0602_03

[ref16] Çi̇çek İ. Çeri̇ V. (2021). Olumlu Çocukluk Yaşantıları Ölçeği: Türkçe Geçerlik ve Güvenirlik Çalışması. Humanist. Perspective 3, 643–659. doi: 10.47793/hp.980149

[ref17] Çiçek İ. Yıldırım M. (2025). Exploring the impact of family conflict on depression, anxiety and sleep problems in Turkish adolescents: the mediating effect of social connectedness. J. Psychol. Couns. Schools 35, 130–146. doi: 10.1177/20556365251331108

[ref18] Coşkun F. Metin Emre A. Hıra Selen A. T. (2024). Klinik Bir Örneklemde Ergenlerde Akran Zorbalığı Sıklığı, Zorbalık Özellikleri ve Zorbalığın Algılanan Sosyal Destekle İlişkisi. Uludağ Üniv. Tıp Fak. Derg. 50, 281–287. doi: 10.32708/uutfd.1518866

[ref19] Coyle S. Cipra A. Rueger S. Y. (2021). Bullying types and roles in early adolescence: latent classes of perpetrators and victims. J. Sch. Psychol. 89, 51–71. doi: 10.1016/j.jsp.2021.09.003, PMID: 34836576

[ref20] Crandall A. Broadbent E. Stanfill M. Magnusson B. M. Novilla M. L. B. Hanson C. L. . (2020). The influence of adverse and advantageous childhood experiences during adolescence on young adult health. Child Abuse Negl. 108:104644. doi: 10.1016/j.chiabu.2020.104644, PMID: 32795716

[ref21] Davila S. A. Martinez A. Medrano A. S. (2025). Navigating familial stressors and material needs: examining resilience and family cohesion as protective factors for rural Mexican adolescents. Cultur. Divers. Ethnic Minor. Psychol. doi: 10.1037/cdp0000754, PMID: 40323811

[ref22] Delgado E. Serna C. Martínez I. Cruise E. (2022). Parental attachment and peer relationships in adolescence: a systematic review. Int. J. Environ. Res. Public Health 19:1064. doi: 10.3390/ijerph19031064, PMID: 35162088 PMC8834420

[ref23] Eilert D. W. Buchheim A. (2023). Attachment-related differences in emotion regulation in adults: a systematic review on attachment representations. Brain Sci. 13:884. doi: 10.3390/brainsci13060884, PMID: 37371364 PMC10296607

[ref24] Elmore A. L. Crouch E. (2020). The Association of Adverse Childhood Experiences with anxiety and depression for children and youth, 8 to 17 years of age. Acad. Pediatr. 20, 600–608. doi: 10.1016/j.acap.2020.02.012, PMID: 32092442 PMC7340577

[ref25] Feeney J. A. Noller P. Hanrahan M. (1994). “Assessing adult attachment” in Attachment in adults: clinical and developmental perspectives. eds. Sperling M. B. Berman W. H. (Guilford Press).

[ref26] Fok C. C. T. Allen J. Henry D. People Awakening Team (2014). The brief family relationship scale: a brief measure of the relationship dimension in family functioning. Assessment 21, 67–72. doi: 10.1177/1073191111425856 22084400 PMC3292682

[ref27] Galaitsi S. E. Wells E. Zemba V. Blue S. Cato C. Wood M. . (2024). Family and community interventions: a meta-analysis on family and community resilience, cohesion, and functioning. Int. J. Disaster Risk Reduct. 110:104597. doi: 10.1016/j.ijdrr.2024.104597

[ref28] Güvendeğer Doksat N. Demirci Ciftci A. (2016). Bağlanma ve Yaşamdaki İzdüşümleri. Arşiv Kaynak Tarama Dergisi 25, 489–501. doi: 10.17827/aktd.253561

[ref29] Hayes A. F. (2018). Introduction to mediation, moderation, and conditional process analysis: A regression-based approach. 2nd Edn: Guilford Press.

[ref30] Hayes A. F. (2022). Introduction to mediation, moderation, and conditional process analysis: a regression-based approach. 3rd Edn: The Guilford Press.

[ref31] Iwanaga K. Blake J. Yaghmaian R. Umucu E. Chan F. Brooks J. M. . (2018). Preliminary validation of a short-form version of the attachment style questionnaire for use in clinical rehabilitation counseling research and practice. Rehabil. Counsel. Bull. 61, 205–216. doi: 10.1177/0034355217709477

[ref32] Joo Y. S. Lee W. K. (2020). Does living in a chaotic home predict adolescent delinquency? A moderated mediation model of impulsivity and school connectedness. Child Youth Serv. Rev. 119:105617. doi: 10.1016/j.childyouth.2020.105617

[ref33] Kars M. P. Peker A. (2025). Ergenlerin Aile İçi İlişkileri ile Aile Bütünlüğü Arasındaki İlişkiler. Sosyal Politika Çalışmaları Dergisi 2025, 99–129. doi: 10.21560/spcd.vi.1634139

[ref34] Keizer R. Helmerhorst K. O. W. Van Rijn-van Gelderen L. (2019). Perceived quality of the mother–adolescent and father–adolescent attachment relationship and adolescents’ self-esteem. J. Youth Adolesc. 48, 1203–1217. doi: 10.1007/s10964-019-01007-0, PMID: 30887261 PMC6525131

[ref35] Keskin G. Çam O. (2008). Ergenlerin ruhsal durumları ve anne baba tutumları ile bağlanma stilleri arasındaki ilişkinin incelenmesi. Anadolu Psikiyiyatri Derg. 9, 139–147.

[ref36] Kline R. B. (2023). Principles and practice of structural equation modeling: Guilford Publications.

[ref37] Kohlhoff J. Lieneman C. Cibralic S. Traynor N. McNeil C. B. (2022). Attachment-based parenting interventions and evidence of changes in toddler attachment patterns: an overview. Clin. Child. Fam. Psychol. Rev. 25, 737–753. doi: 10.1007/s10567-022-00405-4, PMID: 35982272 PMC9622506

[ref38] Kokkinos C. M. Algiovanoglou I. Voulgaridou I. (2019). Emotion regulation and relational aggression in adolescents: parental attachment as moderator. J. Child Fam. Stud. 28, 3146–3160. doi: 10.1007/s10826-019-01491-9

[ref39] Lewis K. N. McKelvey L. M. (2025). Positive childhood experiences support emotional and behavioral health in middle childhood: longitudinal mediation of adverse childhood experiences. Child Abuse Negl. 163:107320. doi: 10.1016/j.chiabu.2025.107320, PMID: 39985881

[ref40] Li M. (2025). The relationship between parent-child conflict and non-suicidal self-injury: a moderated mediating model. Int. J. Ment. Health Promot. 27, 89–95. doi: 10.32604/ijmhp.2024.057223

[ref41] Lin L.-Y. Chien Y.-N. Chen Y.-H. Wu C.-Y. Chiou H.-Y. (2022). Bullying experiences, depression, and the moderating role of resilience among adolescents. Front. Public Health 10:872100. doi: 10.3389/fpubh.2022.872100, PMID: 35692326 PMC9174695

[ref42] Luo A. Kong W. He H. Li Y. Xie W. (2022). Status and influencing factors of social media addiction in Chinese medical care professionals: a cross-sectional survey. Front. Psychol. 13:888714. doi: 10.3389/fpsyg.2022.888714, PMID: 35572263 PMC9097152

[ref43] Mahmood S. Lakatos K. Kalo Z. (2025). Bullying-induced trauma symptomatology among adolescents in Bangladesh: the mediating role of attachment styles. Prev. Med. Rep. 53:103034. doi: 10.1016/j.pmedr.2025.103034, PMID: 40206843 PMC11979516

[ref44] Neville R. D. Madigan S. Fortuna L. R. Porche M. V. Lakes K. D. (2025). Bidirectional associations between parent–child conflict and child and adolescent mental health. J. Am. Acad. Child Adolesc. Psychiatry. doi: 10.1016/j.jaac.2024.12.010 39848441

[ref45] Özada A. Duyan V. (2018). Ebeveyn-Çocuk İlişkisi ve Zorbalık. Turk. J. Fam. Med. Prim. Care 12, 49–55. doi: 10.21763/tjfmpc.399941

[ref46] Özen D. Ş. Aktan T. (2010). Bağlanma ve Zorbalık Sisteminde Yer Alma: Başa Çıkma Stratejilerinin Aracı Rolü. Türk Psikol. Derg. 25, 101–113.

[ref47] Perron S. (2013). Family attachment, family conflict, and delinquency in a sample ofrural youthrural youth (master’s theses and capstones. Durham, NH, United States: University of New Hampshire.

[ref48] Pu Q. Luo Y. Niu L. Luo R. Yang Z. Chen F. (2025). Associations between household chaos and mental health in adolescents: the role of parent-adolescent relationship and adolescent resilience. Child Youth Serv. Rev. 173:108312. doi: 10.1016/j.childyouth.2025.108312

[ref49] Qu C. Jia X. Zhu H. (2025). Profiles of parent-child attachment and peer attachment among adolescents and associations with internalizing problems. Int. J. Ment. Health Promot. 27, 401–420. doi: 10.32604/ijmhp.2025.061059

[ref50] Qu G. Ma S. Liu H. Han T. Zhang H. Ding X. . (2022). Positive childhood experiences can moderate the impact of adverse childhood experiences on adolescent depression and anxiety: results from a cross-sectional survey. Child Abuse Negl. 125:105511. doi: 10.1016/j.chiabu.2022.105511, PMID: 35078091

[ref51] Rahal D. Fosco G. M. (2025). Positive well-being and dampened emotional reactivity to daily family conflict and family cohesion. Child Dev. 96, 797–811. doi: 10.1111/cdev.14206, PMID: 39665301 PMC11868686

[ref52] Rokach A. Clayton S. (2023). “Bullying” in Adverse childhood experiences and their life-long impact (Elsevier), 163–180.

[ref53] Roman N. V. Balogun T. V. Butler-Kruger L. Danga S. D. Therese De Lange J. Human-Hendricks A. . (2025). Strengthening family bonds: a systematic review of factors and interventions that enhance family cohesion. Soc. Sci. 14:371. doi: 10.3390/socsci14060371

[ref54] Şanli M. E. Cicek I. Yıldırım M. Çeri V. (2024). Positive childhood experiences as predictors of anxiety and depression in a large sample from Turkey. Acta Psychol. 243:104170. doi: 10.1016/j.actpsy.2024.104170, PMID: 38301406

[ref55] Scholtes C. M. Cederbaum J. A. (2024). Examining the relative impact of adverse and positive childhood experiences on adolescent mental health: a strengths-based perspective. Child Abuse Negl. 157:107049. doi: 10.1016/j.chiabu.2024.107049, PMID: 39303436

[ref56] Sevi̇nç G. Şener Kilinç T. (2018). Güvenli Bağlanma ve Benlik Kurguları ile Üniveristeye Başlayan Öğrencilerin Uyumu Arasındaki İlişki. Eğitimde Kuram ve Uygulama 14, 306–324. doi: 10.17244/eku.377465

[ref9001] Shaw T. Dooley J. J. Cross D. Zubrick S. R. Waters S. (2013). The Forms of Bullying Scale (FBS): validity and reliability estimates for a measure of bullying victimization and perpetration in adolescence. Psychological assessment, 25, 1045–1057. 23730831 10.1037/a0032955

[ref57] Spruit A. Goos L. Weenink N. Rodenburg R. Niemeyer H. Stams G. J. . (2020). The relation between attachment and depression in children and adolescents: a multilevel Meta-analysis. Clin. Child. Fam. Psychol. Rev. 23, 54–69. doi: 10.1007/s10567-019-00299-9, PMID: 31392452 PMC7000490

[ref58] Vegas M. I. (2025). Family functioning and aggression among Spanish adolescents. Examining the roles of family cohesion, family flexibility, family communication and family satisfaction. J. Fam. Ther. 47:e12478. doi: 10.1111/1467-6427.12478

[ref59] Wang L. Jiang S. Zhou Z. Fei W. Wang W. (2024). Online disinhibition and adolescent cyberbullying: a systematic review. Child Youth Serv. Rev. 156:107352. doi: 10.1016/j.childyouth.2023.107352

[ref60] Williams K. (2011). Bullying behaviors and attachment styles (electronic theses and dissertations. Statesboro, GA: Georgia Southern University.

[ref61] Yavrutürk A. R. (2025). Akran Zorbalığının Psikolojik Temelleri ve Çözüm Önerileri. Sosyal Politika ve Sosyal Hizmet Çalışmaları Dergisi 6, 123–144. doi: 10.61861/spshcd.1705465

[ref62] Yıldırım M. Çiçek İ. Öztekin G. G. Aziz I. A. Hu J. (2023). Associations between problematic social media use and psychological adjustment in Turkish adolescents: mediating roles of family relationships. Int. J. Ment. Health Addict. 23, 811–829. doi: 10.1007/s11469-023-01138-3

[ref63] Yılmaz E. N. (2024). Liseli Ergenlerde Akran Zorbalığı: Bağlanma Stilleri ve Zorbalık Davranışı. Ankara, Turkiye: Akademisyen Kitabevi.

[ref64] Yöndem Z. D. Totan T. (2007). Ergenlerde Zorbalığın Anne Baba ve Akran İlişkileri Açısından İncelenmesi. Ege Eğitim Dergisi 8, 53–68.

[ref65] Yu B. Tong J. Guo C. (2025). Association between parental phubbing and adolescents’ depression: roles of family cohesion and resilience. Child Youth Serv. Rev. 169:108082. doi: 10.1016/j.childyouth.2024.108082

[ref66] Zhang Y. Ma S. Liu Y. Kong F. Zhen Z. (2023). Functional integration of anterior insula related to meaning in life and loneliness. J. Affect. Disord. 338, 10–16. doi: 10.1016/j.jad.2023.05.067, PMID: 37244540

